# Increased Contractile Function of Human Saphenous Vein Grafts Harvested by “No-Touch” Technique

**DOI:** 10.3389/fphys.2017.01135

**Published:** 2018-01-12

**Authors:** Lene P. Vestergaard, Leila Benhassen, Ivy S. Modrau, Frank de Paoli, Ebbe Boedtkjer

**Affiliations:** ^1^Department of Biomedicine, Aarhus University, Aarhus, Denmark; ^2^Department of Cardiothoracic Surgery, Aarhus University Hospital, Aarhus, Denmark

**Keywords:** coronary artery disease, coronary artery bypass grafting, perivascular adipose tissue, saphenous vein, vasoconstriction

## Abstract

Saphenous vein grafts are the most common conduits used for coronary artery bypass grafting (CABG); however, no more than 60% of vein grafts remain open after 10 years and graft failure is associated with poor clinical outcome. The “no-touch” harvesting technique—where a sheet of perivascular tissue is retained around the vein—improves graft patency to over 80% after 16 years of follow-up, but the mechanism for the improved patency rate is unclear. In this study, we investigated acute functional differences between vein grafts harvested conventionally and by “no-touch” technique and explored the importance of perivascular tissue for reducing surgical trauma, minimizing excessive distension, and releasing vasoactive paracrine factors. Segments of human saphenous veins were obtained from CABG surgery and their functional properties investigated by isometric and isobaric myography. We found a broad diameter-tension relationship for human saphenous veins, with peak capacity for active tension development at diameters corresponding to transmural pressures around 60 mmHg. Across the investigated transmural pressure range between 10 and 120 mmHg, maximal tension development was higher for “no-touch” compared to conventionally harvested saphenous veins. Contractile responses to serotonin, noradrenaline, and depolarization induced with elevated extracellular [K^+^] were significantly larger for saphenous veins harvested by “no-touch” compared to conventional technique. Conventional vein grafts are routinely pressurized manually in order to test for leaks; however, avoiding this distension procedure did not change the acute contractile function of the conventionally excised saphenous veins. In contrast, even though surgical removal of perivascular tissue during conventional harvesting was associated with a substantial decrease in force development, removal of perivascular tissue by careful dissection under a stereomicroscope only marginally affected contractile responses of veins harvested by “no-touch” technique. In conclusion, we show that saphenous veins harvested by “no-touch” technique have greater contractile capacity than veins harvested by conventional technique. The different capacity for smooth muscle contraction is not due to vasoactive substances released by the perivascular tissue. Instead, we propose that the larger tension development of saphenous veins harvested by “no-touch” technique reflects reduced surgical damage, which may have long-term consequences that contribute to the superior graft patency.

## Introduction

The long-term success of coronary artery bypass grafting (CABG) is negatively influenced by graft failure (Lopes et al., 2012; Blachutzik et al., 2016). Patency of conventionally harvested saphenous vein grafts is 80–90% 1 year after the CABG procedure and decreases to no more than 60% after 10 years (Campeau et al., 1979; Fitzgibbon et al., 1996; Harskamp et al., 2013a; de Vries et al., 2016). The left internal thoracic artery is considered first choice conduit because of prolonged high patency (Otsuka et al., 2013) but equivalent results (e.g., 83% patency after 16 years) can be achieved with saphenous vein grafts harvested by so-called “no-touch” technique (Souza et al., 2001, 2006; Johansson et al., 2009; Samano et al., 2015). When “no-touch” vein grafts are excised, a sheet of perivascular tissue is retained around the vein (Souza, 1996). Suggestions have been made that improved integrity of the endothelial and medial smooth muscle cell layers (Ahmed et al., 2004; Vasilakis et al., 2004; Loesch et al., 2006), higher expression and activity of endothelial NO synthase (Tsui et al., 2001; Dashwood et al., 2009), greater density of vasa vasorum (Dreifaldt et al., 2011), and paracrine release of vasoactive substances from the perivascular tissue (Fernández-Alfonso et al., 2017) contribute to the superior patency of “no-touch” compared to conventional saphenous vein grafts. Nevertheless, the impact of perivascular tissue and harvesting techniques on the pathophysiology of graft occlusion is still not well-understood.

Vein grafts used for CABG surgery experience dramatic changes in mechanical forces—in particular increased transmural pressure and linear velocity of the blood—when they are transplanted from the venous to the arterial circulation. Elevated shear stress and circumferential wall stress of arteries compared to veins of equal caliber are important factors that influence vessel wall remodeling and neointima formation (Fitts et al., 2014). Increased physiological shear stress—which is typically 10–70 dynes/cm^2^ in arteries compared to 1–5 dynes/cm^2^ in veins (dela Paz and D'Amore, 2009; Fitts et al., 2014)—usually leads to outward remodeling. However, damaged endothelial cell layer caused by excessive physical forces can lead to endothelial dysfunction or even allow shear forces to act directly on the smooth muscle cells (Fry, 1969; Fitts et al., 2014). The mechanotransduction pathways and pathophysiological consequences of increased mechanical stress remain controversial and may depend on the flow pattern, but effects of shear stress on smooth muscle cell phenotype (i.e., shift from contractile to synthetic phenotype) and proliferation have been demonstrated and may contribute to inward remodeling and luminal obstruction of transplanted vein grafts (Kipshidze et al., 2004; Shi and Tarbell, 2011). It is well-accepted that surgical procedures and graft characteristics affect the rate of graft failure (Harskamp et al., 2013b), and it is expected that long-term patency is improved if the transplanted graft is functionally and structurally intact at the time of anastomosis.

In addition to providing structural support, the secretory activity of perivascular tissue can have functional implications for the vascular wall (Fernández-Alfonso, 2004). Crosstalk between perivascular tissue and resistance arteries in many vascular beds contributes to metabolic regulation of blood flow whereby local perfusion is adjusted to the metabolic demand. Proposed paracrine factors released from perivascular tissue include NO, leptin, adiponectin, prostanoids, angiotensin 1–7, and hydrogen sulfide (Simonsen and Boedtkjer, 2016), which in addition to vasomotor effects may modify the structure of the blood vessel wall, for instance, through changes in cell migration, proliferation, and apoptosis. Perivascular cardiomyocyte-rich tissue surrounding coronary arteries modifies both vasocontraction and—relaxation (Aalbaek et al., 2015). Even though the cause-effect relationship is still unclear, the relevance of altered crosstalk between perivascular tissue and cells of the vascular wall for disease is supported by altered anti-contractile influences of perivascular tissue in models of type 2 diabetes (Bonde et al., 2017), obesity (Yudkin et al., 2005; Greenstein et al., 2009; Aghamohammadzadeh et al., 2013), inflammation (Bhattacharya et al., 2013), and hypertension (Li et al., 2013).

There is evidence for structural damage to the vascular wall of conventionally harvested vein grafts (Ahmed et al., 2004; Loesch et al., 2006). However, functional evidence of altered vasomotor performance has not been provided and consequences of different harvesting techniques for the vasomotor function of excised grafts have not been systematically evaluated. In the current study, we hypothesized that harvest of saphenous vein grafts by “no-touch” technique improves the immediate contractile function because (a) the adherent perivascular tissue releases vasoactive paracrine factors that modify smooth muscle contractions or (b) the atraumatic “no-touch” surgical technique reduces damage and preserves smooth muscle contractile capacity. We find that saphenous vein grafts harvested by “no-touch” technique have increased capacity for force generation and that this greater contractile function is unrelated to acute release of paracrine factors from the perivascular tissue. We propose that improved graft viability with associated greater dynamic range of tone regulation contributes to the superior long-term patency of “no-touch” vein grafts used for CABG surgery.

## Materials and methods

“No-touch” vein harvest technique was implemented at Aarhus University Hospital in 2011, 5 years prior to the present study. All conduits were harvested by the same team of cardiac surgeons experienced in both “no-touch” and conventional harvest techniques. The study was carried out in accordance with Danish legislation and the protocol approved by the Mid-Jutland Regional Committee on Health Research Ethics (enquiry no. 167/2017). According to Danish legislation, written informed consent was not required because the procedures involve excess resected tissue from a surgical procedure where all post-surgical tissue and data handling was anonymized.

### Conventional harvesting technique

The saphenous vein was exposed by a longitudinal incision through the skin, the perivascular tissue stripped from the adventitial layer, and all side branches divided between clips or ligatures. The saphenous vein was removed and distended manually with a 10-mL syringe containing heparin-saline-−0.90% (weight/volume) aqueous NaCl solution containing 50 IU/mL heparin—to check for leaks. Attention was paid not to overdistend the vein. After distension, the vein graft was stored in heparin-saline at room temperature until anastomosis.

### “No-touch” harvesting technique

The course of the saphenous vein was delineated with a marker on the overlying skin using B-mode ultrasonography and a longitudinal incision was made through the skin until the saphenous vein was visualized. The saphenous vein was excised with adherent perivascular tissue along the whole circumference and all side branches were divided between clips or ligatures. At the surgeons discretion, one of two approaches was now chosen: the vein was either (a) stored at room temperature in papaverine-containing, heparinized autologous blood solution −60 mL blood added 1,000 IU heparin and 60 mg papaverine—until it was connected to the aortic cannula and perfused through the arterial line, or (b) manually distended and stored at room temperature in the papaverine-containing, heparinized autologous blood solution until anastomosis. Attention was paid not to overdistend the vein.

### Isometric myography

The vein segments investigated for contractile function were stored only very briefly (typically, a few minutes) in the above-mentioned heparin-saline or autologous blood solution. The vein segments were then transferred to chilled physiological saline solutions (PSS) and transported (~20 min duration) on ice to the laboratory at Aarhus University for analysis of contractile function. Two millimeter long segments of saphenous vein grafts were mounted in 4-channel wire myographs (610 M; DMT, Aarhus Denmark) on 100-μm tungsten pins either with the amount of associated perivascular tissue provided by the surgeons or after the perivascular tissue was removed by microdissection under a stereomicroscope (Zeiss, Germany). The time for transport and subsequent handling of veins was similar for veins harvested by conventional and “no-touch” technique. Diameter-tension relationships were established by first stretching the relaxed veins under continuous force recordings to diameters corresponding to fixed transmural pressures, and then trigger maximal active tension with 125 mM extracellular K^+^ and 10 μM serotonin. Concentration-response relationships to noradrenaline, serotonin, and extracellular K^+^ were established in veins that—when fully relaxed—were normalized to an internal diameter corresponding to a transmural pressure of either 20 mmHg (representative of the venous circulation) or 100 mmHg (representative of the arterial circulation; Mulvany and Halpern, 1977). When the vein segments were set to new diameters by moving the myograph pins apart, stretch-induced damage was avoided by first allowing time for full relaxation in Ca^2+^-free PSS containing 10 μM sodium nitroprusside (SNP). Under these conditions, the equivalent transmural pressure was calculated based on LaPlace's law.

### Isobaric myography

Saphenous vein segments were microdissected free from surrounding perivascular tissue and mounted with ligatures on two silicone tubes in a pressure myograph (110P; DMT). The inner diameter *(ID)* of the vein segments was visualized and recorded with MyoVIEW 3.2 software (DMT). Vasoconstriction was evaluated during cumulative application of serotonin at transmural pressures of 20 and 100 mmHg. The fully relaxed diameter (*ID*_passive_) was recorded by exposing the veins to Ca^2+^-free PSS containing 5 mM EGTA and 10 μM papaverine. For each condition, we calculated: Active tone = (*ID*_passive_-*ID*_active_)/*ID*_passive_, where all diameters correspond to the same transmural pressure.

### Composition of solutions

The PSS used for evaluation of venous contractile function had the following composition (in mM): NaCl 119, CaCl_2_ 1.6, KCl 4.7, MgSO_4_ 1.7, NaHCO_3_ 22, KH_2_PO_4_ 1.18, EDTA 0.026, and glucose 5.5. PSS with high [K^+^] was produced by equimolar substitution of KCl for NaCl. All solutions were titrated to pH 7.4 at 37°C after vigorous bubbling with a gas mixture of 5% CO_2_/balance air.

### Statistics

Data are expressed as mean ± SEM; *n* equals number of patients. Probability (*P*) values <0.05 were considered statistically significant. One or two vessel segments from each patient were investigated for each condition; if two vessel segments from the same patient were tested, the average value was used to represent that patient in subsequent statistical analyses to ensure that values within each group were independent. Diameter-tension and concentration-response relationships were fitted to second order polynomial and sigmoidal functions, respectively, and compared between experimental conditions with extra sum-of-squares *F*-tests. The effects of two variables on a third variable, measured multiple times for each patient, were evaluated by repeated measures two-way ANOVA followed by Sidak's post-tests. Statistical analyses were performed using Microsoft Excel 2010 and GraphPad Prism 7.03 software.

## Results

### Saphenous veins have broad diameter-tension relationships and maximal active tension development is increased in “no-touch” veins

**Figure 1:**
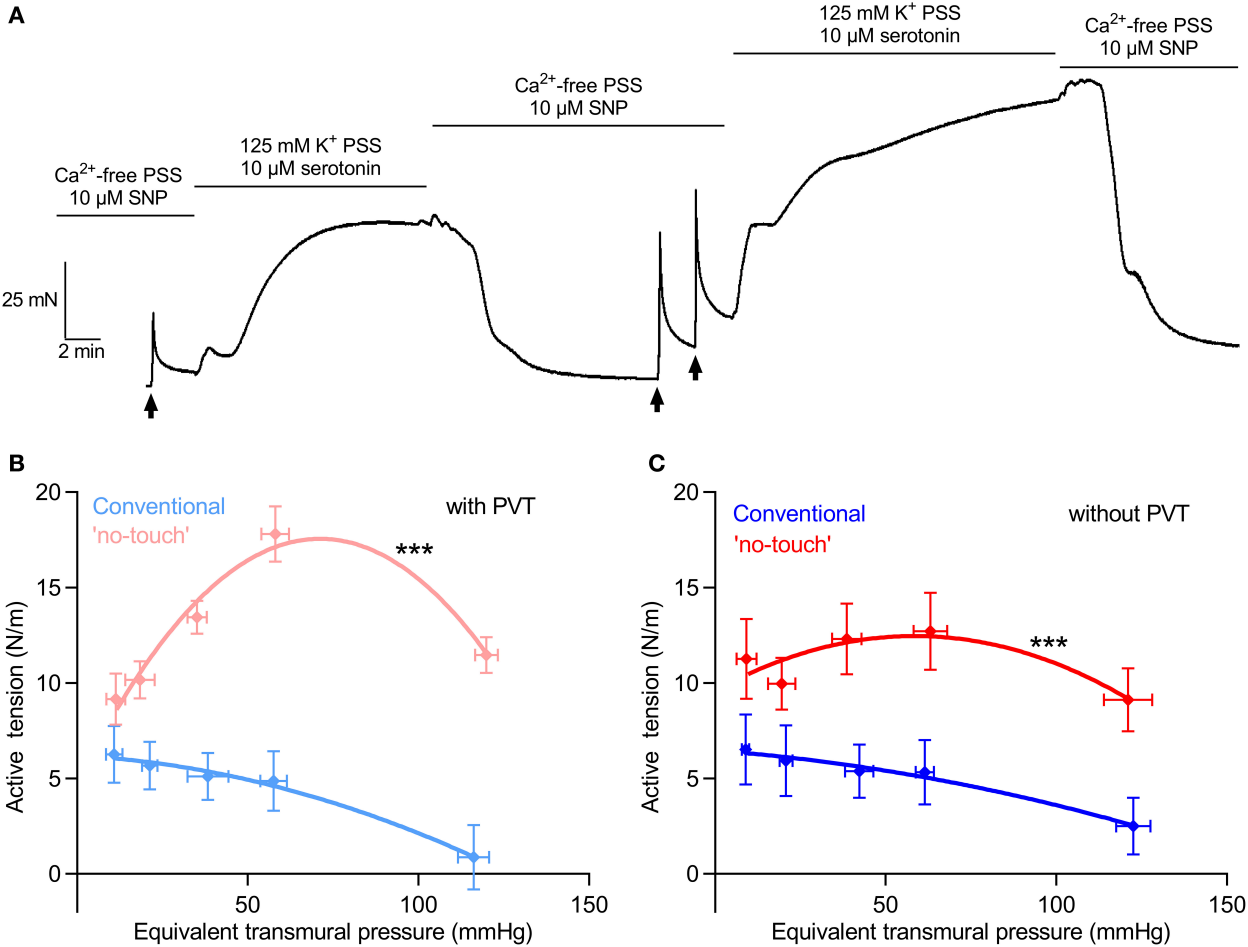
Saphenous veins have a broad diameter-tension relationship and the capacity for maximal tension development is increased in “no-touch” veins. **(A)** Original force trace showing the protocol used for establishing the diameter-tension relationship for saphenous vein grafts. The arrows indicate time points where the micrometer screw is turned to stretch the vein to a larger diameter. The protocol was repeated multiple times to cover the pressure interval between 10 and 120 mmHg. **(B,C)**. Maximal active tension development plotted as function of the equivalent passive transmural pressure for vein graft segments surrounded by the amount of perivascular tissue (PVT) retained by the surgeon (**B**, *n* = 8–11) and after removal of PVT by careful dissection under a stereomicroscope (**C**, *n* = 8–11). The curves are results of second order polynomial fits and we compare them by extra sum-of-squares *F*-tests. ^***^P < 0.05 *vs*. Conventional.

Initially, we determined the relationship between the degree of passive stretch and the capacity of the harvested vein grafts for maximal active tension development. The saphenous vein segments were relaxed and stepwise stretched in Ca^2+^-free PSS with 10 μM SNP; and at each progressively larger diameter, the capacity for maximal active force development—after washout of SNP and return of extracellular Ca^2+^–was tested by exposure to 125 mM extracellular K^+^ and 10 μM serotonin. An original trace showing the protocol is provided in Figure 1A. The passive diameters were converted to equivalent transmural pressures using LaPlace's law. As shown in Figure 1B, the “no-touch” saphenous veins showed peak active tension development at diameters equivalent to around 60 mmHg, and relatively small decreases in capacity for active tension development were observed when the passive diameter was increased or decreased within the broad interval corresponding to transmural pressures between 20 and 120 mmHg. Striking differences in maximal active tension development were observed between veins harvested by “no-touch” and conventional technique: at its peak, maximal tension development was 3-fold larger in “no-touch” than conventional vein grafts (Figure 1B).

To investigate whether the difference in maximal tension development was due to vasoactive paracrine factors released from the perivascular tissue, we next carefully removed the perivascular tissue from the “no-touch” vein grafts by microdissection under a stereomicroscope. Any small amount of perivascular tissue remaining on the conventionally harvested vein grafts was removed in a similar manner. We saw a small, yet significant (*P* < 0.05, extra sum-of-squares *F*-test), reduction in vasocontractile capacity when the perivascular tissue was carefully removed by microdissection (compare Figures 1B,C). Notably, the difference in maximal active tension development between the veins harvested by “no-touch” and conventional technique was also evident after the perivascular tissue had been removed (Figure 1C). These findings support that possible vasomotor effects of paracrine factors released from the perivascular tissue are not the main causes of the acute difference in vasocontractile function observed between veins harvested by “no-touch” and conventional technique.

### Isometric vasocontraction at “venous” and “arterial” transmural pressures is not affected by microdissection of perivascular tissue

**Figure 2:**
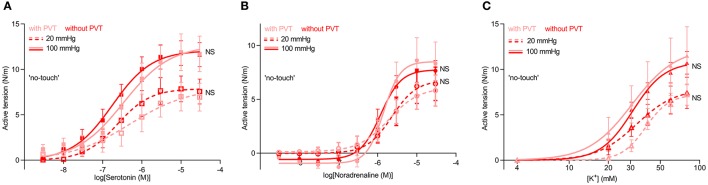
Vasocontraction of saphenous veins harvested by “no-touch” technique is not affected by removal of perivascular tissue (PVT) by microdissection. **(A–C)** Vasocontractile responses to serotonin (**A**, *n* = 8–9), noradrenaline (**B**, *n* = 8–9), and elevated extracellular [K^+^] (**C**, *n* = 7–8). Experiments involving elevated extracellular [K^+^] were performed in the presence of 1 μM phentolamine in order to inhibit effects of noradrenaline released from perivascular nerve endings in response to depolarization. 20 and 100 mmHg refer to the equivalent transmural pressures at which the veins were investigated. The curves are the results of least-squares fits to sigmoidal functions, and we compare them using extra sum-of-squares *F*-tests. NS: not significantly different vs. with PVT.

To further explore whether paracrine factors released from the perivascular tissue surrounding saphenous veins have vasomotor effects, veins harvested by “no-touch” technique were each cut into two segments: one was investigated as supplied from the surgeon whereas the perivascular tissue was carefully removed from the other by microdissection under a stereomicroscope. The veins were mounted for isometric investigation and stretched to internal diameters corresponding to typical mean venous and arterial transmural pressures of 20 and 100 mmHg, respectively. The acute removal of perivascular tissue had no significant effect on the concentration-dependent vasoconstrictor response elicited by serotonin (Figure 2A) or the sympathetic neurotransmitter noradrenaline (Figure 2B). Responses to elevated extracellular [K^+^]—applied in order to bypass receptor activation and investigate the response to direct depolarization of vascular smooth muscle cells—also did not differ between “no-touch” veins with and without perivascular tissue (Figure 2C). Responses to elevated extracellular [K^+^] were evaluated in the presence of 1 μM α-receptor antagonist phentolamine in order to block effects of noradrenaline released from perivascular nerve endings in response to depolarization. These findings are consistent with the diameter-tension relationships (Figure 1) and support that the differences in vasocontractile function between veins harvested by conventional and “no-touch” technique are not explained by acute vasomotor effects of paracrine factors released from the perivascular tissue.

### Isometric vasocontraction is increased in veins harvested by “no-touch” technique

**Figure 3:**
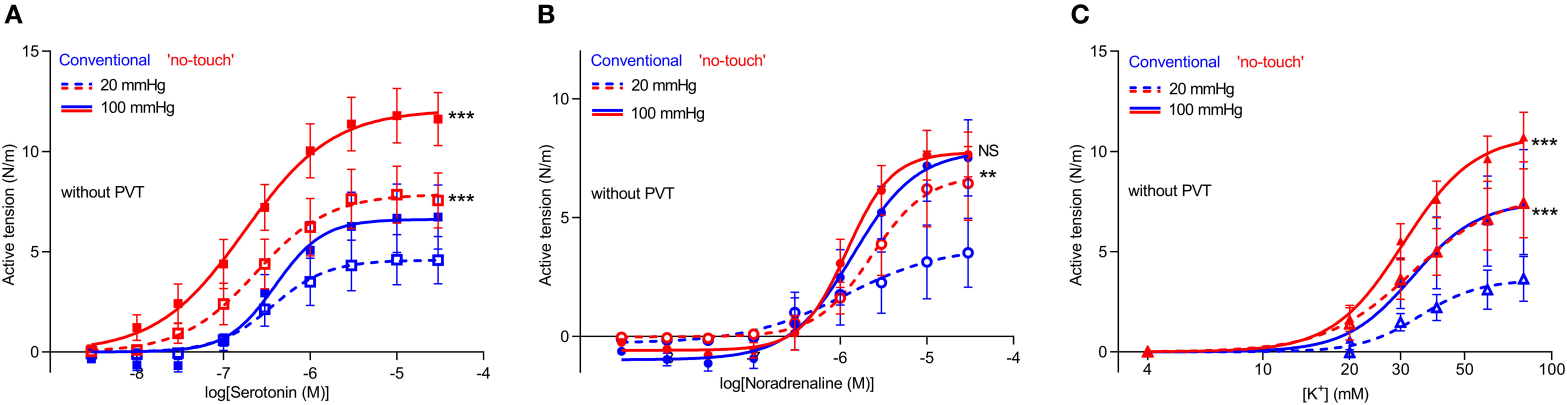
Vasocontraction in response to noradrenaline, serotonin and depolarization with elevated [K^+^] is increased in saphenous veins harvested by “no-touch” technique. **(A–C)** Vasocontractile responses to serotonin (**A**, *n* = 6–9), noradrenaline (**B**, *n* = 6–9), and elevated extracellular [K^+^] (**C**, *n* = 7–8). 20 and 100 mmHg refer to the equivalent transmural pressures at which the veins were investigated. Experiments involving elevated extracellular [K^+^] were performed in the presence of 1 μM phentolamine in order to inhibit effects of noradrenaline released from perivascular nerve endings in response to depolarization. The data relating to “no-touch” veins are replotted from Figure 2 to ease interpretations. The curves are the results of least-squares fits to sigmoidal functions, and we compare them using extra sum-of-squares *F*-tests. ^**^P < 0.01, ^***^P < 0.001, NS: not significantly different vs. Conventional.

We next microdissected and mounted saphenous vein segments harvested by “no-touch” and conventional technique in wire myographs to compare their contractile function. The concentration-dependent tension development in response to serotonin (Figure 3A) and noradrenaline (Figure 3B) was greater in veins harvested by “no-touch” technique than in veins harvested by conventional technique; although for noradrenaline, the difference between the harvesting procedures was evident only when veins were normalized to a transmural pressure of 20 mmHg. Saphenous veins harvested by “no-touch” technique also produced stronger contractions than veins harvested by conventional technique when exposed to stepwise increments in extracellular [K^+^] (Figure 3C).

### Endothelium-dependent vasorelaxation is modest in vein grafts and unaffected by harvest procedure

**Figure 4:**
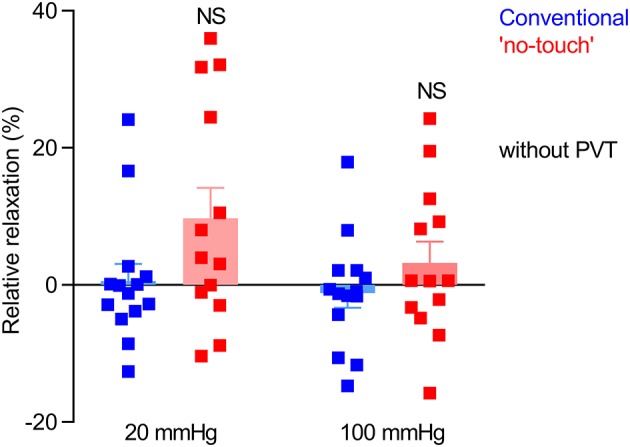
Endothelium-dependent vasorelaxation is modest and variable in vein grafts and not significantly different between veins harvested by conventional and “no-touch” technique. The vein grafts (*n* = 13–14) were stretched to an internal diameter corresponding to a transmural pressure of 20 or 100 mmHg, pre-contracted with 10 μM serotonin, and exposed to 10 μM methacholine. The data are compared by repeated measures two-way ANOVA followed by Sidak's post-test. NS: not significantly different vs. Conventional.

In order to evaluate the function of the endothelium, we next tested vasorelaxation of serotonin-pre-contracted veins in response to methacholine. When veins were pre-contracted with 10 μM serotonin, they relaxed on average <10% upon application of methacholine (Figure 4), which is in agreement with previous observations (Wise et al., 2015). Although there was a tendency toward improved vasorelaxation of veins harvested by “no-touch” compared to conventional technique, this did not reach statistical significance (*P* = 0.09), see Figure 4.

### Isobaric vasoconstriction is increased in veins harvested by “no-touch” technique

**Figure 5:**
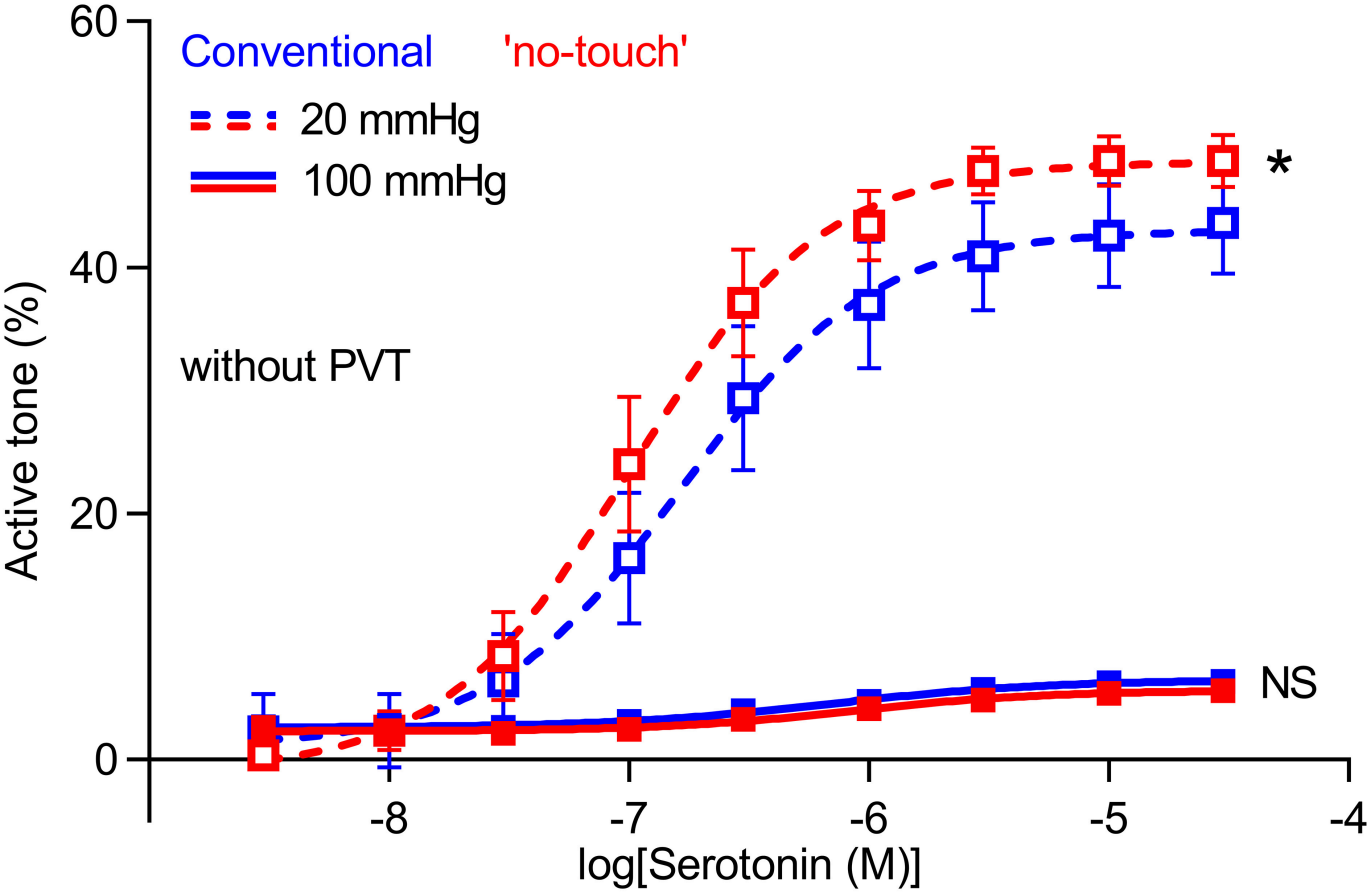
Isobaric vasoconstriction is increased in “no-touch” vein grafts. Veins harvested by “no-touch” (*n* = 7) and conventional (*n* = 7) technique were constricted with serotonin at transmural pressures of 20 and 100 mmHg. The curves are the results of least-squares fits to sigmoidal functions, and we compare them using extra sum-of-squares *F*-tests. ^*^P < 0.05, NS: not significantly different vs. Conventional.

To further support the greater vasomotor responses of “no-touch” saphenous veins, we next mounted veins—microdissected free from perivascular tissue—in pressure myographs and exposed them to transmural pressures of 20 or 100 mmHg. At 20 mmHg, veins harvested by “no-touch” technique produced stronger vasoconstriction in response to serotonin than veins harvested by conventional technique (Figure 5). Active tone development was much attenuated at a transmural pressure of 100 mmHg; and at this higher pressure, no difference was observed between veins excised by the two different harvesting techniques (Figure 5).

### Avoiding manual distension of conventionally harvested vein grafts does not recover tension development

**Figure 6:**
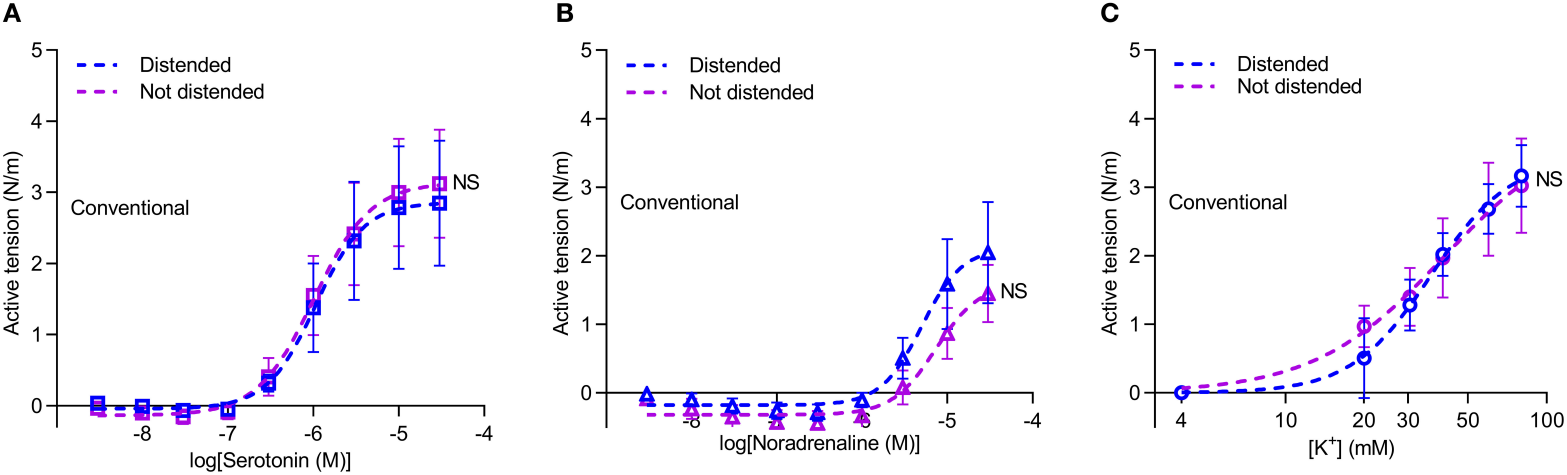
Avoiding distension of conventional vein grafts does not lead to larger contractions. **(A–C)** Vasocontraction in response to serotonin (**A**, *n* = 7), noradrenaline (**B**, *n* = 6) and elevated extracellular [K^+^] (**C**, *n* = 6). Arteries were normalized to an internal diameter corresponding to a transmural pressure of 20 mmHg. Experiments involving elevated extracellular [K^+^] were performed in the presence of 1 μM phentolamine in order to inhibit effects of noradrenaline released from perivascular nerve endings in response to depolarization. The curves are the results of least-squares fits to sigmoidal functions, and we compare them using extra sum-of-squares *F*-tests. NS: not significantly different vs. Distended Conventional veins.

As part of the harvest procedure, vein grafts are pressurized to check for leaks. We investigated if the reduced vasocontraction of conventionally harvested veins compared to veins harvested by “no-touch” technique is explained by damage during this manual distension, which could be exacerbated in veins lacking the structural support from the perivascular tissue. To explore this possibility, we investigated a subset of veins harvested by conventional technique, except that one segment of the vein was not exposed to manual distension. Avoiding manual graft distension did not increase contractile responses of conventionally harvested veins during stimulation with serotonin (Figure 6A), noradrenaline (Figure 6B) or elevated extracellular [K^+^] (Figure 6C). Based on these findings, we conclude that damage during manual distension is not responsible for the lower vasocontraction of conventionally harvested vein grafts.

## Discussion

A series of well-designed and well-conducted randomized clinical trials from a single center in Sweden with up to 16 years of angiographic follow-up provides strong evidence of superior patency rates of “no-touch” compared to conventional saphenous vein grafts (Souza et al., 2001, 2006; Johansson et al., 2009; Samano et al., 2015). The current study evaluates whether the acute vasomotor function of “no-touch” saphenous vein grafts differs from that of conventionally harvested vein grafts and was conducted in order to identify functional properties that might help to understand the improved patency.

Our studies show that saphenous veins harvested by “no-touch” technique have greater contractile capacity than equivalent grafts harvested by conventional technique (Figures 1, 3, 5). The stronger contractions—evident under both isometric (Figures 1, 3) and isobaric (Figure 5) experimental conditions—are observed in response to the vascular agonists serotonin and noradrenaline (Figures 3A,B, 5) and also when smooth muscle cell membrane depolarization is induced by elevating extracellular [K^+^] (Figure 3C).

Vasoactive effects of paracrine factors released from perivascular tissue have been described for multiple arterial preparations from humans and rodents (Gollasch, 2012) and in rat inferior vena cava (Lu et al., 2011). In contrast, we find that removal of the perivascular tissue surrounding human saphenous vein segments only has marginal acute effects on their contractile properties (Figures 1, 2) suggesting that the perivascular adipose tissue surrounding saphenous veins has very little net vasomotor effect. This is in congruence with a rather loose association between the perivascular tissue and the saphenous veins compared, for instance, to coronary arteries. Nevertheless, additional studies are required to determine whether the perivascular tissue is important for long-term graft patency.

Because the greater capacity for vasocontraction of “no-touch” relative to conventional vein grafts is evident also after the perivascular tissue has been removed by microdissection (Figures 1, 2, 3, 5), our studies suggest that the sheath of perivascular tissue is important for atraumatic excision and handling rather than as a source of paracrine secretion. It is commonly observed that stretch or forceful dilation of contracted blood vessels damages their function; and to minimize this effect, vasodilators (e.g., papaverine) are routinely used during CABG graft harvesting (He et al., 1993). In the investigated saphenous vein segments, we observed that if veins were not fully relaxed during the normalization procedure or during the passive stretch component of the diameter-tension protocol, they immediately lost contractile function. Thus, our findings are consistent with the perivascular tissue being critical for permitting the surgeon to avoid directly grasping the vein with surgical instruments that can lead to cell damage and associated vasospasm (Souza and Samano, 2016; Fernández-Alfonso et al., 2017), and protecting against direct damage induced by sharp dissection during stripping of conventional vein grafts.

The proposed importance of the perivascular tissue as a mechanical shield against surgical handling suggests that the amount of retained perivascular tissue can be reduced to a thin sheath, which will minimize the size of the surgical wound on the harvest site and the risk of wound infection. Infection is a challenge of the open surgical techniques compared to endoscopic harvesting techniques (Kopjar et al., 2016; Souza and Samano, 2016). Still, clinical verification of the amount of perivascular tissue necessary for improved long-term graft function and clinical outcome is necessary as there is currently no direct evidence that the capacity for vein graft contraction is causatively related to its long-term patency.

Venous transmural pressure in healthy humans is typically around 20 mmHg but can increase to around 60 mmHg during sustained upright posture or in patients with insufficient venous valve function (Koster et al., 2013). Maximal active tension development at transmural pressures higher than the physiological level (Figure 1) provides basis for a Starling-like mechanism that will limit vein compliance during combined venous build-up of blood and activation of venous smooth muscle cells. The increased active tension development will support local intravascular pressure increases that can facilitate return of blood to the heart through the valve-containing venous circulation.

Previous studies have shown that the storage conditions used between harvest and implantation can affect the quality of CABG vein grafts. In particular, endothelium-dependent vasorelaxation has been found improved by storage in buffered salt solutions (Wise et al., 2015). In the current study, the excised grafts were stored for a few minutes in the storage solution used for the surgical procedure (i.e., heparin-saline or heparinized papaverine-containing autologous blood solution) before they were transferred to a buffered salt solution (i.e., PSS) that was used for transport, dissection, and experiments. Using buffered salt solution for storage and handling, we avoid that variation in storage conditions differentially affect the function of vein grafts harvested by conventional and “no-touch” technique.

Although we primarily consider the enhanced contractile capacity of “no-touch” vein grafts an important sign of improved viability, the greater capacity for vasocontraction also provides enhanced ability for dynamic regulation of precapillary resistance, local perfusion, and capillary pressure. A greater viability of the medial vascular smooth muscle cells of “no-touch” vein grafts is consistent with previous histological investigations (Ahmed et al., 2004; Vasilakis et al., 2004; Loesch et al., 2006). Because cell damage in the vascular wall is associated with structural disintegration and platelet deposition, it is expected that improved graft viability in the acute phase can reduce long-term graft failure.

In conclusion, we demonstrate for the first time that saphenous vein grafts harvested by “no-touch” technique have stronger vasocontractile capacity than vein grafts harvested by conventional technique. In the “no-touch” veins, the increase in active tension development at transmural pressures higher than the physiological level provides basis for a Starling-like mechanism that could limit build-up of blood and counteract edema formation. Whereas, acute vasomotor effects of paracrine factors released from the perivascular tissue were negligible, our findings support that the atraumatic “no-touch” surgical technique improves graft viability, which is consistent with the greater long-term patency.

## Author contributions

IM, FdP, and EB: conceived the project; LV and LB: performed and analyzed experiments; All authors contributed to the design of experiments and interpretation of data; EB: wrote the manuscript; All authors revised the manuscript and accepted the final version.

### Conflict of interest statement

The authors declare that the research was conducted in the absence of any commercial or financial relationships that could be construed as a potential conflict of interest.

## References

[B1] Aalbaek F. Bonde L. Kim S. Boedtkjer E. (2015). Perivascular tissue inhibits rho-kinase-dependent smooth muscle Ca^2+^ sensitivity and endothelium-dependent H_2_S signalling in rat coronary arteries. J. Physiol. 593, 4747–4764. 10.1113/JP271006 26350036 PMC4626546

[B2] Aghamohammadzadeh R. Greenstein A. S. Yadav R. Jeziorska M. Hama S. Soltani F.. (2013). Effects of bariatric surgery on human small artery function: evidence for reduction in perivascular adipocyte inflammation and the restoration of normal anticontractile activity despite persistent obesity. J. Am. Coll. Cardiol. 62, 128–135. 10.1016/j.jacc.2013.04.027 23665100 PMC3791397

[B3] Ahmed S. R. Johansson B. L. Karlsson M. G. Souza D. S. R. Dashwood M. R. Loesch A. (2004). Human saphenous vein and coronary bypass surgery: ultrastructural aspects of conventional and “no-touch” vein graft preparations. Histol. Histopathol. 19, 421–433. 10.14670/HH-19.421 15024703

[B4] Bhattacharya I. Drägert K. Albert V. Contassot E. Damjanovic M. Hagiwara A.. (2013). Rictor in perivascular adipose tissue controls vascular function by regulating inflammatory molecule expression. Arterioscler. Thromb. Vasc. Biol. 33, 2105–2111. 10.1161/ATVBAHA.112.301001 23868942

[B5] Blachutzik F. Achenbach S. Troebs M. Roether J. Nef H. Hamm C.. (2016). Angiographic findings and revascularization success in patients with acute myocardial infarction and previous coronary bypass grafting. Am. J. Cardiol. 118, 473–476. 10.1016/j.amjcard.2016.05.040 27328951

[B6] Bonde L. Shokouh P. Jeppesen P. B. Boedtkjer E. (2017). Crosstalk between cardiomyocyte-rich perivascular tissue and coronary arteries is reduced in the zucker diabetic fatty rat model of type 2 diabetes mellitus. Acta Physiol. 219, 227–238. 10.1111/apha.12685 27042951

[B7] Campeau L. Lespérance J. Hermann J. Corbara F. Grondin C. M. Bourassa M. G. (1979). Loss of the improvement of angina between 1 and 7 years after aortocoronary bypass surgery: correlations with changes in vein grafts and in coronary arteries. Circulation 60, 1–5. 10.1161/01.CIR.60.2.1 312705

[B8] Dashwood M. R. Savage K. Tsui J. C. Dooley A. Shaw S. G. Fernandez Alfonso M. S.. (2009). Retaining perivascular tissue of human saphenous vein grafts protects against surgical and distension-induced damage and preserves endothelial nitric oxide synthase and nitric oxide synthase activity. J. Thorac. Cardiovasc. Surg. 138, 334–340. 10.1016/j.jtcvs.2008.11.060 19619776

[B9] de Vries M. R. Simons K. H. Jukema J. W. Braun J. Quax P. H. A. (2016). Vein graft failure: from pathophysiology to clinical outcomes. Nat. Rev. Cardiol. 13, 451–470. 10.1038/nrcardio.2016.76 27194091

[B10] dela Paz N. G. D'Amore P. A. (2009). Arterial versus venous endothelial cells. Cell Tissue Res. 335, 5–16. 10.1007/s00441-008-0706-5 18972135 PMC4105978

[B11] Dreifaldt M. Souza D. S. R. Loesch A. Muddle J. R. Karlsson M. G. Filbey D.. (2011). The “no-touch” harvesting technique for vein grafts in coronary artery bypass surgery preserves an intact vasa vasorum. J. Thorac. Cardiovasc. Surg. 141, 145–150. 10.1016/j.jtcvs.2010.02.005 20381817

[B12] Fernández-Alfonso M. S. Gil-Ortega M. Aranguez I. Souza D. Dreifaldt M. Somoza B.. (2017). Role of PVAT in coronary atherosclerosis and vein graft patency: friend or foe? Br. J. Pharmacol. 174, 3561–3572. 10.1111/bph.13734. 28150299 PMC5610150

[B13] Fernández-Alfonso M. S. (2004). Regulation of vascular tone. Hypertension 44, 255–256. 10.1161/01.HYP.0000140056.64321.f9 15289465

[B14] Fitts M. K. Pike D. B. Anderson K. Shiu Y. T. (2014). Hemodynamic shear stress and endothelial dysfunction in hemodialysis access. Open Urol. Nephrol. J. 7, 33–44. 10.2174/1874303X01407010033 25309636 PMC4189833

[B15] Fitzgibbon G. M. Kafka H. P. Leach A. J. Keon W. J. Hooper G. D. Burton J. R. (1996). Coronary bypass graft fate and patient outcome: angiographic follow-up of 5,065 grafts related to survival and reoperation in 1,388 patients during 25 years. J. Am. Coll. Cardiol. 28, 616–626. 10.1016/0735-1097(96)00206-9 8772748

[B16] Fry D. L. (1969). Certain histological and chemical responses of the vascular interface to acutely induced mechanical stress in the aorta of the dog. Circ. Res. 24, 93–108. 10.1161/01.RES.24.1.93 5763742

[B17] Gollasch M. (2012). Vasodilator signals from perivascular adipose tissue. Br. J. Pharmacol. 165, 633–642. 10.1111/j.1476-5381.2011.01430.x 21486288 PMC3315036

[B18] Greenstein A. S. Khavandi K. Withers S. B. Sonoyama K. Clancy O. Jeziorska M.. (2009). Local inflammation and hypoxia abolish the protective anticontractile properties of perivascular fat in obese patients. Circulation 119, 1661–1670. 10.1161/CIRCULATIONAHA.108.821181 19289637

[B19] Harskamp R. E. Lopes R. D. Baisden C. E. de Winter R. J. Alexander J. H. (2013a). Saphenous vein graft failure after coronary artery bypass surgery: pathophysiology, management, and future directions. Ann Surg. 257, 824–833. 10.1097/SLA.0b013e318288c38d 23574989

[B20] Harskamp R. E. Williams J. B. Hill R. C. de Winter R. J. Alexander J. H. Lopes R. D. (2013b). Saphenous vein graft failure and clinical outcomes: toward a surrogate end point in patients following coronary artery bypass surgery? Am. Hear. J. 165, 639–643. 10.1016/j.ahj.2013.01.019 23622900 PMC4089872

[B21] He G. W. Rosenfeldt F. L. Angus J. A. (1993). Pharmacological relaxation of the saphenous vein during harvesting for coronary artery bypass grafting. Ann. Thorac. Surg. 55, 1210–1217. 10.1016/0003-4975(93)90036-H 8494433

[B22] Johansson B. L. Souza D. S. R. Bodin L. Filbey D. Bojö L. (2009). No touch vein harvesting technique for CABG improves the long-term clinical outcome. Scand. Cardiovasc. J. 43, 63–68. 10.1080/14017430802140104 18609044

[B23] Kipshidze N. Dangas G. Tsapenko M. Moses J. Leon M. B. Kutryk M.. (2004). Role of the endothelium in modulating neointimal formation. J. Am. Coll. Cardiol. 44, 733–739. 10.1016/j.jacc.2004.04.048 15312851

[B24] Kopjar T. Dashwood M. R. Biocina B. (2016). Is local wound infection rate more important than long-term graft patency in coronary artery bypass grafting? J. Thorac. Cardiovasc. Surg. 151, 275. 10.1016/j.jtcvs.2015.09.066 26699777

[B25] Koster M. Amann-Vesti B. R. Husmann M. Jacomella V. Meier T. O. Jeanneret C.. (2013). Non-invasive pressure measurement of the great saphenous vein in healthy controls and patients with venous insufficiency. Clin. Hemorheol. Microcirc. 54, 325–332. 10.3233/CH-131737. 23686088

[B26] Li R. Andersen I. Aleke J. Golubinskaya V. Gustafsson H. Nilsson H. (2013). Reduced anti-contractile effect of perivascular adipose tissue on mesenteric small arteries from spontaneously hypertensive rats: role of Kv7 channels. Eur. J. Pharmacol. 698, 310–315. 10.1016/j.ejphar.2012.09.026 23059186

[B27] Loesch A. Dashwood M. R. Souza D. S. R. (2006). Does the method of harvesting the saphenous vein for coronary artery bypass surgery affect venous smooth muscle cells? iNOS immunolabelling and ultrastructural findings. Int. J. Surg. 4, 20–29. 10.1016/j.ijsu.2005.11.002 17462310

[B28] Lopes R. D. Mehta R. H. Hafley G. E. Williams J. B. Mack M. J. Peterson E. D.. (2012). Relationship between vein graft failure and subsequent clinical outcomes after coronary artery bypass surgery. Circulation. 125, 749–756. 10.1161/CIRCULATIONAHA.111.040311 22238227 PMC3699199

[B29] Lu C. Zhao A. X. Gao Y.-J. Lee R. M. (2011). Modulation of vein function by perivascular adipose tissue. Eur. J. Pharmacol. 657, 111–116. 10.1016/j.ejphar.2010.12.028 21236250

[B30] Mulvany M. J. Halpern W. (1977). Contractile properties of small arterial resistance vessels in spontaneously hypertensive and normotensive rats. Circ. Res. 41, 19–26. 10.1161/01.RES.41.1.19 862138

[B31] Otsuka F. Yahagi K. Sakakura K. Virmani R. (2013). Why is the mammary artery so special and what protects it from atherosclerosis? Ann. Cardiothorac Surg. 2, 519–526. 10.3978/j.issn.2225-319X.2013.07.06 23977631 PMC3741888

[B32] Samano N. Geijer H. Liden M. Fremes S. Bodin L. Souza D. (2015). The no-touch saphenous vein for coronary artery bypass grafting maintains a patency, after 16 years, comparable to the left internal thoracic artery: a randomized trial. J. Thorac. Cardiovasc. Surg. 150, 880–888. 10.1016/j.jtcvs.2015.07.027 26282605

[B33] Shi Z.-D. Tarbell J. M. (2011). Fluid flow mechanotransduction in vascular smooth muscle cells and fibroblasts. Ann. Biomed. Eng. 39, 1608–1619. 10.1007/s10439-011-0309-2 21479754 PMC3184546

[B34] Simonsen U. Boedtkjer E. (2016). New roles of factors from perivascular tissue in regulation of vascular tone. Acta. Physiol. 216, 159–162. 10.1111/apha.12620 26495823

[B35] Souza D. Samano N. (2016). Long-term patency versus leg wound healing in coronary artery bypass surgery: surgical aspects of the no-touch harvesting technique. J. Thorac. Cardiovasc. Surg. 151, 276. 10.1016/j.jtcvs.2015.10.025 26699779

[B36] Souza D. S. R. Bomfim V. Skoglund H. Dashwood M. R. Borowiec J. W. Bodin L.. (2001). High early patency of saphenous vein graft for coronary artery bypass harvested with surrounding tissue. Ann. Thorac. Surg. 71, 797–800. 10.1016/S0003-4975(00)02508-X 11269454

[B37] Souza D. S. Johansson B. Bojö L. Karlsson R. Geijer H. Filbey D.. (2006). Harvesting the saphenous vein with surrounding tissue for CABG provides long-term graft patency comparable to the left internal thoracic artery: results of a randomized longitudinal trial. J. Thorac. Cardiovasc. Surg. 132, 373.e5–378.e5. 10.1016/j.jtcvs.2006.04.002 16872965

[B38] Souza D. (1996). A new no-touch preparation technique: technical notes. Scand. J. Thoracic. Cardiovasc. Surg. 30, 41–44. 10.3109/14017439609107239 8727856

[B39] Tsui J. C. Souza D. S. Filbey D. Bomfim V. Dashwood M. R. (2001). Preserved endothelial integrity and nitric oxide synthase in saphenous vein grafts harvested by a “no-touch” technique. Br. J. Surg. 88, 1209–1215. 10.1046/j.0007-1323.2001.01855.x 11531869

[B40] Vasilakis V. Dashwood M. R. Souza D. S. R. Loesch A. (2004). Human saphenous vein and coronary bypass surgery: scanning electron microscopy of conventional and “no-touch” vein grafts. Vasc. Dis. Prev. 1, 133–139. 10.2174/1567270043405204

[B41] Wise E. S. Hocking K. M. Eagle S. Absi T. Komalavilas P. Cheung-Flynn J.. (2015). Preservation solution impacts physiologic function and cellular viability of human saphenous vein graft. Surgery 158, 537–546. 10.1016/j.surg.2015.03.036 26003912 PMC4492846

[B42] Yudkin J. S. Eringa E. Stehouwer C. D. (2005). “Vasocrine” signalling from perivascular fat: a mechanism linking insulin resistance to vascular disease. Lancet 365, 1817–1820. 10.1016/S0140-6736(05)66585-3 15910955

